# Integrated QSAR study for inhibitors of hedgehog signal pathway against multiple cell lines:a collaborative filtering method

**DOI:** 10.1186/1471-2105-13-186

**Published:** 2012-07-31

**Authors:** Jun Gao, Dongsheng Che, Vincent W Zheng, Ruixin Zhu, Qi Liu

**Affiliations:** 1 College of Life Science and Biotechnology, Tongji University, Shanghai, 200092, China; 2 College of Information Engineering, Shanghai Maritime University, Shanghai, 201306, China; 3 Department of Computer Science, East Stroudsburg University, East Stroudsburg, PA, USA; 4 Advanced Digital Sciences Center, Illinois at Singapore Pte, Singapore, Singapore

## Abstract

**Background:**

The Hedgehog Signaling Pathway is one of signaling pathways that are very important to embryonic development. The participation of inhibitors in the Hedgehog Signal Pathway can control cell growth and death, and searching novel inhibitors to the functioning of the pathway are in a great demand. As the matter of fact, effective inhibitors could provide efficient therapies for a wide range of malignancies, and targeting such pathway in cells represents a promising new paradigm for cell growth and death control. Current research mainly focuses on the syntheses of the inhibitors of cyclopamine derivatives, which bind specifically to the Smo protein, and can be used for cancer therapy. While quantitatively structure-activity relationship (QSAR) studies have been performed for these compounds among different cell lines, none of them have achieved acceptable results in the prediction of activity values of new compounds. In this study, we proposed a novel collaborative QSAR model for inhibitors of the Hedgehog Signaling Pathway by integration the information from multiple cell lines. Such a model is expected to substantially improve the QSAR ability from single cell lines, and provide useful clues in developing clinically effective inhibitors and modifications of parent lead compounds for target on the Hedgehog Signaling Pathway.

**Results:**

In this study, we have presented: (1) a collaborative QSAR model, which is used to integrate information among multiple cell lines to boost the QSAR results, rather than only a single cell line QSAR modeling. Our experiments have shown that the performance of our model is significantly better than single cell line QSAR methods; and (2) an efficient feature selection strategy under such collaborative environment, which can derive the commonly important features related to the entire given cell lines, while simultaneously showing their specific contributions to a specific cell-line. Based on feature selection results, we have proposed several possible chemical modifications to improve the inhibitor affinity towards multiple targets in the Hedgehog Signaling Pathway.

**Conclusions:**

Our model with the feature selection strategy presented here is efficient, robust, and flexible, and can be easily extended to model large-scale multiple cell line/QSAR data. The data and scripts for collaborative QSAR modeling are available in the Additional file 1.

## Background

The Hedgehog Signaling Pathway plays an important role in regulating embryonic development in vertebrates, and it is highly conserved from flies to humans [1][2][3][4].The pathway name comes from a polypeptide ligand called Hedgehog (Hh), which is an intercellular signaling molecule in Drosophila. In Drosophila, the mutation of the gene in the Hedgehog Signaling Pathway gives rise to an unusual spiky-haired phenotype [1]. The misregulation of such pathways has been directly associated with a variety of inherited and sporadic diseases [4][5][6]. The key role of the Hedgehog Signaling Pathway in the cell differentiation, growth, and proliferation makes it an excellent candidate in drug discovery, and thus targeting such pathway in cells represents a promising new paradigm for cell growth and death control.

**Figure 1:**
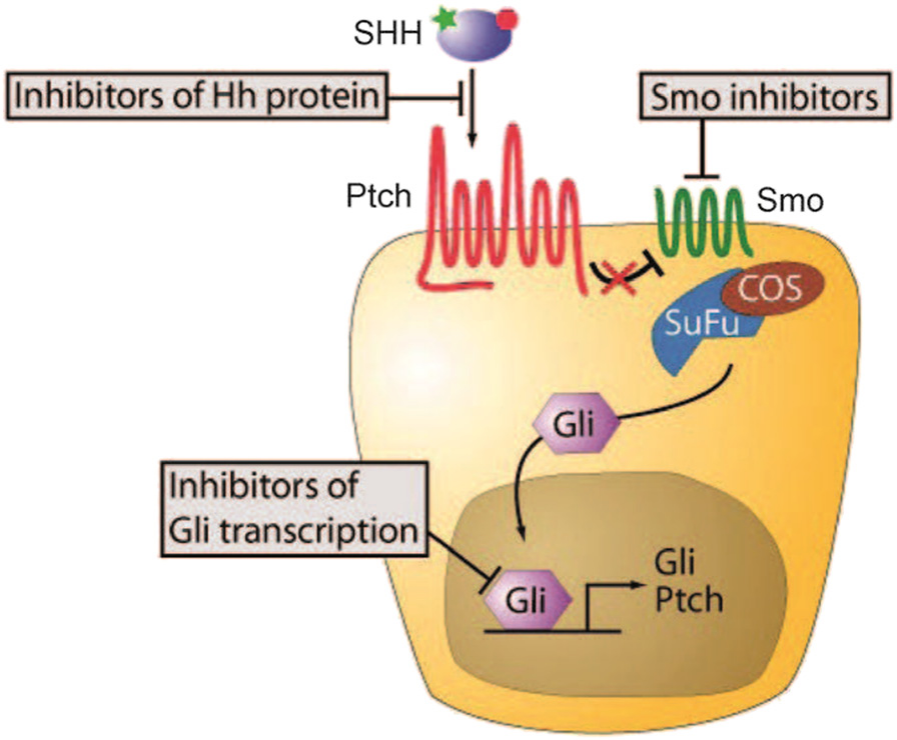
Components of the Hedgehog (Hh) Signaling Pathway and molecular sites targeted by Hh pathway inhibitors.

The Hedgehog Signal Pathway is composed of four important components: Sonic Hedgehog, Patched, Smoothened and Gli transcription factors [3] (Figure 1). The functional Hh protein secreted from the membranes of the producing cells and initiates the Hh signaling cascade upon binding to the 12-pass transmembrane receptor Patched (Ptch). In the absence of an Hh ligand, the Patched receptor inhibits the activity of the downstream seven-pass transmembrane receptor Smoothened (Smo), which resembles G-protein-coupled receptors (GPCRs) in general topology. Active Smo then signals via a cytosolic complex of proteins including Suppressor of Fused (SuFu), and the cascade culminates by triggering activation of the glioma (Gli) family of transcription factors and their translocation to the nucleus. This activation results in the expression of specific genes that promote cell proliferation and differentiation [3].

The causal relationship between the activation of Hedgehog Signaling Pathway and oncogenesis has driven cancer researchers in the direction of finding specific inhibitors of hedgehog signaling, since this will provide efficient therapies to a wide range of malignancies [1,2]. To date, several druggable nodes within the pathway have been identified. Assays implanted on various cell lines have shown that small molecules were able to alter the activity of these targets. Among them, murine cell lines such as NIH 3 T3, TM3h12, and C3H10T1/2 have been used [2]. While current cell lines allow the measurement of the inhibitory effects of compounds on the Hh pathway, they, however, provide little or no information about the specific underlying targets. To the best of our knowledge, only specific Smoothened inhibitors have been identified. Among them, the well-known BODIPY–cyclopamine, which is a fluorescent derivative of the naturally occurring Smo antagonist cyclopamine, binds specifically to cells expressing the Smo protein. This is one of the small chemical compounds that specifically inhibit Smoothened in the Hedgehog Signaling Pathway[2]. In our previous study [7], we have performed several quantitatively structure-activity relationship (QSAR) studies for cyclopamine derivatives in multiple cell lines, and such study could reveal useful clues in developing clinically effective drugs and modifications of parent lead compounds for cancer therapy.

Recently, our partners have synthesized 93 cyclopamine derivatives and their activities were tested against four different cell lines (BxPC-3, NCI-H446, SW1990 and NCI-H157) respectively [7][8]. Based on these experimental data, a systematical QSAR investigation was carried out by incorporation of various statistic modelings and different molecular descriptors [7]. However, there are still several issues remain to be solved, which we believe that solving such problems will greatly enhance the understanding of inhibitors on Hedgehog Signaling Pathway, as well as the development of novel QSAR methodology. We describe the two major problems below:

(1) In our previous QSAR study, for specific cell lines, the activities were categorized into a binary classification under a naïve Bayesian model, and we obtained relatively acceptable QSAR results. However, no matter what kinds of statistical models or 2D descriptors were tested, low testing correlation coefficients were found when numeric activities were used. This may be due to the inherent noise existed in experimental activity measurement, or the relatively small number of training data used for a specific cell line. Due to our compound data tested against multiple cell lines to evaluate their activities, we hypotheses that such information can be integrated to improve the QSAR results rather than only a single cell line QSAR modeling. Such investigation will be extremely useful for the scenario that a small number of compound activities are measured under different experimental conditions (such as different cell lines, targets, assays etc.), and will provide novel insights on the integration of existing information, avoiding repeatable laborious work in drug discovery. In addition, such a study may also lead to novel computational models for integrated QSAR modeling, which is closely related to multi-task QSAR modeling [9][10], Multi-Assay-Based QSAR modeling [11] , and Multi-target QSAR study [12].

(2) Due to the existence of compound activity data against multiple cell lines, how can we integrate such information to derive more robust and efficient feature selection strategies for compound modification under such “collaborative” multi-cell line environment? That is, can we derive the commonly important features related to the entire given cell lines for compound description, while in the meantime present their specific contributions to a specific cell-line? This issue is closely related to the first one, but tougher to be solved since it needs much more domain knowledge.

Inspired by these two problems, we aim to develop an efficient integrated QSAR model for inhibitors of Hedgehog Signal Pathway against multiple cell lines. This type of model has been used for information retrieval in social network, i.e. collaborative filtering [13][14], and it has been widely applied by the web companies such as Google, Amazon. Dumitru Erhan etc. has pioneered to use the term “collaborative filtering” in multiple target study [15]. Nevertheless, their methodology can be categorized as multiple regression or neural network, and a complex kernel function for similarity measurement is needed. In this study, we will present a collective matrix factorization based collaborative filtering model for integrated QSAR modeling, which is more naturally suitable for QSAR modeling, and scales up well on large dataset. Furthermore, we will also derive a powerful feature selection strategy for collaborative compound design to get more efficient inhibitors of Hedgehog Signal Pathway.

## Methods and materials

### Dataset

93 cyclopamine derivatives with their activities against four different cell lines (BxPC-3, NCI-H446, SW1990 and NCI-H157) were obtained from our previous work [7]. The compound activity is measured by *PK*_*i*_, as defined in the following Cheng-Prusoff equation [16]:

(1) $$PK_{i}=-\log\frac{IC_{50}}{1+\frac{\left[L\right]}{K_{D}}}$$

(*L* is the concentration of free radioligand used and *K*_*D*_ is its equilibrium dissociation constant for the receptor [16])

Where *IC*_*50*_ (half maximal inhibitory concentration) is a measure of the effectiveness of a compound in inhibiting biological or biochemical function. More specifically, it indicates how much of a particular drug or other substance (inhibitor) needed to inhibit a given biological process (or component of a process, i.e. an enzyme, cell, cell receptor or microorganism) by half. In our study, the data are formulated as a data matrix *X*. Note that the collective matrix factorization requires the matrix to be non-negative. In our original experiments, we measured the compound affinity under the PK_i_ evaluation system, and the activity values were negative. Since the *PK*_*i*_ measurement is calculated by taking *IC*_*50*_ as the input in equation (1), we can just take the absolute value of the PK_i_ in QSAR modeling , and this will not affect our final results.

### Definitions and Notations

In this paper, the different cell lines and the compounds tested for Hedgehog Signal Pathway will be denoted as *t* and *c* respectively, and their corresponding subscripts denote a specific compound and cell line. Thus, for a specific compound *c*_*i*_, its experimentally activity value (measured as PK_i_ ) against specific cell line *t*_*j*_ is denoted as *x*_*ij*_. We can build a *m* by *n* dimensional matrix *X*, where *m* is the number of the compounds and *n* is the number of cell lines.

Each compound will be represented by a vector of descriptors, denoted as a matrix *Y* with *m* by *r* dimensions, where *m* is the number of compounds and *r* is the length of the corresponding descriptor vector. Similar to our previous study [7], two different molecule descriptors, general descriptor [17] and drug-like index (DLI) [18] will be used for compound representation.

### Collaborative filtering for multiple cell line QSAR modeling

Based on the above definitions, it can be seen that the traditional single cell line QSAR modeling is applied on the data in a specific column of matrix *X*. In this study, we are more interested in incorporating the information from other columns (cell lines) to enhance the performance of the QSAR modeling for a particular column (cell line). This scenario is similar to the recommendation system presented by Electronic retailers and content providers such as Amazon.com and Netflix [14], which make automatic predictions (filtering) of users’ interests by collecting preferences or taste information from many users (collaborating), naturally termed as “collaborative filtering (CF)”.

**Figure 2:**
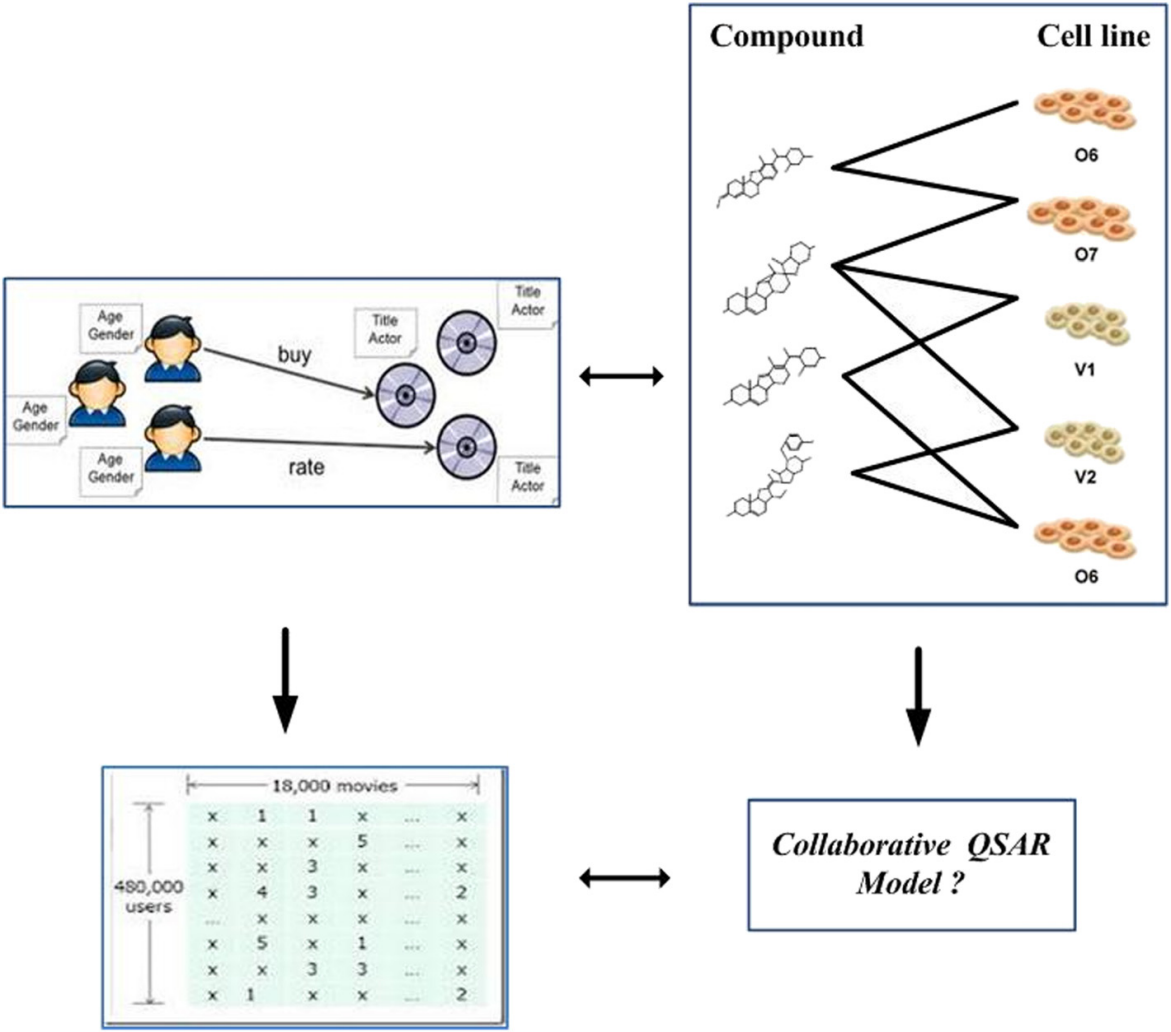
A paralleling comparison between collaborative QSAR modeling and collaborative filtering in social network community.

Formally speaking, in a typical CF scenario, there is a list of *n* users {*u*_*1*_, *u*_*2*_, . . . , *u*_*n*_} and a list of *m* items {*i*_*1*_, *i*_*2*_, . . . , *i*_*m*_}, and each user, *u*_*i*_, has a list of items, *Iu*_*i*_, which the user has rated, or about which their preferences have been inferred through their behaviors. The ratings can either be explicit indication on a 1–5 scale, or implicit indication such as purchases or click-throughs [13]. Such a user-item relationship can be formulated as a matrix, which may be sparse and can have missing values (i.e. users did not give their preferences). The goal of CF is to predict such missing values based on the existed information of users/items to make the reasonable recommendation (Left Panel of Figure 2).

Such a CF scenario is inherently suitable for our multiple cell line QSAR modeling. In our study, the former “cell line- compound” matrix *X* can be viewed as a kind of “item-user” matrix, where “compound” is analogue to “item” and “cell line” is analogue to “user” (Right Panel of Figure 2). The traditional single cell line QSAR modeling uses the data restricted in a specific column of matrix *X* to train and test. From the perspective of machine learning, we just hold part of the data in the column as testing dataset, and use the other part of the data in the column to train a QSAR model. This procedure can be naturally extend to the multiple cell line QSAR modeling under the CF framework, where we can treat the testing data in a specific column as “missing” value and using the remain data from this column as well as the data from other columns to predict such missing values.

### Collective matrix factorization for collaborative filtering in the multiple cell line QSAR modeling

We formulate the multiple cell line QSAR modeling problem as a collaborative filtering problem. There are two existing techniques for solving collaborative filtering, i.e., the neighborhood methods and latent factor models. Neighborhood methods are centered on computing the relationships between items or, alternatively, between users for missing value prediction, while latent factor models characterize both items and users on, say, 20 to 100 factors inferred from the ratings patterns [19][20][21]. Generally, realizations of latent factor models are based on matrix factorization. In its basic form, matrix factorization characterizes both items and users through vectors of factors inferred from item rating patterns. High correlation between item and user factors leads to a recommendation. These methods have become popular in recent years by combining good scalability with predictive accuracy. Thus, we will present a matrix factorization based multiple cell line QSAR modeling method in our study.

Specifically, we have matrix $X\in R_{+}^{m\;x\;n}$, where *X*_*i j*_*,* epresents the activity measurement of compound *i* against specific cell line *j*. Noted that *X* is sparse in a specific column since we will hold part of elements in this column as the testing data (missing values) for QSAR modeling. We use an indicator matrix $I\in R^{m\;x\;n}$ to represent the missing values, where *I*_*ij*_ = 0 if *X*_*ij*_ is missing and *I*_*ij*_ = 1 otherwise.

We denote by $X_{j}.\text{,}\;1\leq \text{i}\leq \text{m and }X._{j}\text{, }1\leq \text{j}\leq \text{n}$ the *i*th row and *j*th column of *X*, which represent the *i*th compound's activities against all the cell lines and the activities of the *j*th cell line for all the compounds, respectively.

In a basic matrix factorization model, we usually seek two low-rank matrices, $U\in {\text{R}}_{+}^{m\times d}$ and $V\in {\text{R}}_{+}^{n\times d}$. The row vector $u_{i}$ and $v_{j}$ represent the low-dimensional representations of compounds and cell lines respectively. We use matrix $\text{U}\times V^{T}$ to approximate the original matrix *X*, thus to fill/predict the missing values. Such matrix factorization can be achieved by solve the following optimization function:

(2) $$\text{min}\;L\left(\text{U},\text{ V}\right)$$

Where

(3) $$\begin{array}{l} L(\text{U},\text{V})=∥I\circ {(X-UV^{T})∥}_{F}^{2}+{\text{λ}}_{1}{∥U∥}_{F}^{2}\\ \;+{\text{λ}}_{2}{∥V∥}_{F}^{2} \end{array}$$

In equation (3), the operator “∘” denotes the entry-wise product. $||*|{|}_{\frac{F}{}}$ denotes the Frobenius norm. The last 2 terms add regularizations to the matrix *U* and *V* by avoiding over-fitting the observed data. The parameters $\lambda_{1}$ and $\lambda_{2}$ control the extent of regularizations, and they are usually determined by cross-validation.

We also have the compound description information as described in matrix *Y* in order to use such auxiliary information to aid a more reasonable reconstruction of matrix *X*. We further presented a collective matrix factorization (CMF)[22] method for multiple cell line QSAR modeling. The CMF method was recently presented by machine learning community [22], and it jointly factorizes multiple matrices simultaneously, assuming that they share several common latent factors. To be more specific, given a compound - cell line matrix $X\in R_{+}^{m\times n}$, and a compound description matrix $Y\in R_{+}^{m\times n}$, we extend the optimization function (2) and (3) to the following:

(4) $$\text{min}\;L\left(\text{U,V,W}\right)$$

Where *L*(U, V, W)

(5) $$\begin{array}{l} =\frac{1}{2}∥I\circ {(X-UV^{T})∥}_{F}^{2}+\frac{{\text{λ}}_{1}}{2}∥Y-UW^{T}∥{}_{F}^{2}\\ \;+\frac{{\text{λ}}_{2}}{2}({∥U∥}_{F}^{2}+{∥V∥}_{F}^{2}+{∥W∥}_{F}^{2}) \end{array}$$

Equation (5) is similar to equation (3), it reconstructs $X\approx UV^{T}$ and $Y\approx UW^{T}$ by sharing the common factor *U*, where $X\in R_{+}^{m\times n}\text{,}\;Y\in R_{+}^{m\times r}\text{,}\;U\in R_{+}^{m\times d},V\in R_{+}^{n\times d}\text{ and }W\in R_{+}^{r\times d}$. *U*, *V* and *W* are low-dimensional matrices with dimensiond ≤min(m,n,r). By solving such optimization function, we can successfully incorporate the information of the multiple cell line compound activities and compound description for a better missing value prediction.

In general, the objective function (5) is not jointly convex to all the variables *U*, *V*,*W*, and we cannot get closed-form solutions for minimizing the objective function. Therefore, we will turn to some numerical method such as gradient descent to get the local optimal solutions. Specifically, we have the gradients as:

(6) $$\nabla_{u}L=\left[I\circ \left(UV^{T}-X\right)\right]V+\lambda_{1}\left(UW^{T}-Y\right)W+\lambda_{2}U$$

(7) $$\nabla_{v}L={\left[I\circ \left(UV^{T}-X\right)\right]}^{T}U+\lambda_{2}V$$

(8) $$\nabla_{w}L={\left[I\circ \left(UW^{T}-Y\right)\right]}^{T}U+\lambda_{2}W$$

After obtaining the gradients, we can use gradient descent to iteratively minimize the objective function. The algorithm for the collective two matrix factorization is given below:

Algorithm 1: collective matrix factorization for multiple cell line QSAR modeling

Input: An incomplete matrix *X* and a complete matrix *Y* , where *X* represents the compound activities in multiple cell lines with missing values in specific column, *Y* represents the compound description matrix.

Output: The complete matrix for *X*.

Begin

1. *t* = 1;

2. While (*t* <*T* and $L_{t}-L_{t+1}>\varepsilon$ do

3. Get the gradients $\nabla_{u}L\text{,}\;\nabla_{v}L\text{,}\;\nabla_{w}L$ by Equation (5)-(7);

4. y = 1;

5. While $\left(L({\text{U}}_{t}-\gamma \nabla_{u}{}_{t}L,{\text{V}}_{t}-\gamma \nabla_{v}{}_{t}L,{\text{W}}_{t}-\gamma \nabla_{w}{}_{t}L)\geq L({\text{U}}_{t},{\text{V}}_{t},{\text{W}}_{t})\right)$ do

6. γ=γ/2;

7. End

8. ${\text{U}}_{t+1}={\text{U}}_{t}-\gamma \nabla_{u}{}_{t}L,{\text{V}}_{t+1}={\text{V}}_{t}-\gamma \nabla_{v}{}_{t}L,{\text{W}}_{t+1}={\text{W}}_{t}-\gamma \nabla_{w}{}_{t}L$

9. t = t + 1;

10. End

11. return X;

End

### Performance measurement

In order to demonstrate the efficiency of collective matrix factorization based multiple cell line QSAR modeling, we compare our approach with two other base line methods, i.e., linear ridge regression and support vector regression (SVR) for single cell line QSAR modeling used in our previous study [7]. For the purpose of equal comparison, we apply the following two testing strategies for each specific cell line: (1). We randomly selected 2/3 of the data to train the linear ridge regression and SVR, and the remaining data as to test these two methods. These two base line methods are compared with collective matrix factorization based QSAR method, where the same testing data (missing values) are predicted based on the original training data for this specific cell line plus the data from other cell lines. The whole procedure was repeated 10 times. (2) In order to evaluate the QSAR model more rigorously and consider the representative ability of the compounds in training dataset, we applied another data partition strategy, i.e., *Diverse Subset* data division method [7], which is commonly used in the chemoinformatics community. Generally speaking, the *Diverse Subset* method ranks compound entries based on diversity. In the procedure of data division, the first entry of the original dataset is taken as a reference and will always be viewed as part of a diverse subset. Then the most “distinct” compound data is assigned #2, and then the most distinct compound to these two is assigned #3 and so on until the required number of diverse compounds is identified or the whole dataset is ranked in diversity order [7]. In this study, we also select 1/3 of the data as testing dataset and the remaining data as the training dataset, while such partition is generated in a *Diverse Subset* way rather than randomly to keep the representative and distinct characteristics of data.

Two classical measurements, i.e., Root mean squared error (RMSE) and squared correlation coefficient (*R*-square) were adopted as the performance evaluations for testing results. The definitions of these statistical parameters are provided as follows:

Root mean squared error (RMSE):

(9) $$\text{RMSE}=\sqrt{\frac{1}{n}}\sum_{i=1}^{n}e_{i}^{2}$$

where *n* is the number of test compounds, $e_{i}=y_{i}-{\hat{y}}_{i}$, is the difference between the observed compound affinity data and the fitted model. $y_{i}$ is the observed compound affinity, ${\hat{y}}_{i}$ is the predicted compound affinity.

Squared correlation coefficient (R2):

(10) $$R^{2}=1-\frac{SS_{\mathrm{err}}}{SS_{\mathrm{tot}}}=1-\frac{\sum_{i}{\left(P_{i}^{\exp}-P_{i}^{\mathrm{calc}}\right)}^{2}}{\sum_{i}{\left(P_{i}^{\exp}-P^{\mathrm{avg}}\right)}^{2}}$$

where $P^{\mathrm{avg}}$ is the average value of $P_{i}^{\exp}$ over the *n* predicted compound affinities.

### Feature selection based on CMF for compound description among multiple cell lines

Under such collaborative QSAR schema, we presented a novel feature selection model for compound descriptions weighting, which is also derived from the content-based recommender systems and collaborative filtering [23]. Basically, we want to quantify the effect of each compound feature against a specific cell line (weighting for intra-cell line) as well as among all the cell lines (weighting for inter-cell line). The final feature weighting is an integration of the two types of weighting, where both specific and the whole cell lines contribute. Such a feature selection strategy is attractive in multiple cell line QSAR modeling, since it can provide useful clues of how to modify chemical compounds to improve their activities for a specific target, or for all given cell lines simultaneously. While the latter one is a key step for multi-target compound design.

Specially, given a compound activity-cell line matrix *X* (*m* by *n*) and compound-feature description matrix *Y* (*m* by *r*), we want to derive a cell line-description feature weighting matrix *Z* (*n* by *r*), where its element *z*_*ij*_ is the weight of a compound feature *j* in cell line *i*. The value of element *z*_*ij*_ is contributed from two sides, i.e. intra-cell line and inter-cell line. The generally procedure for computing a weight for each compound feature is based on (1) the amount of information provided by itself , and (2) the correlation between the compound feature and a specific cell line. Three steps are performed here:

Step 1. Weighting for inter-cell line. For each compound feature *c*_*j*_, an entropy based method is applied to compute the amount of information that it can offer regardless of cell line, as denoted as *H*_*j*_.

Step 2. Weighting for intra-cell line. For each compound feature *c*_*j*_ and a specific cell line *t*_*j*_, the correlation between compound feature and the cell line is calculated. This calculation will depend on the nature of the features (qualitative, quantitative). Two kinds of correlations, i.e., correlation coefficient and contingency coefficient [23] are proposed for quantitative features and qualitative features respectively.

Step 3. Calculation of the final weights. The feature weight is obtained as a result of the product of entropy and degree of dependency.

**Figure 3:**
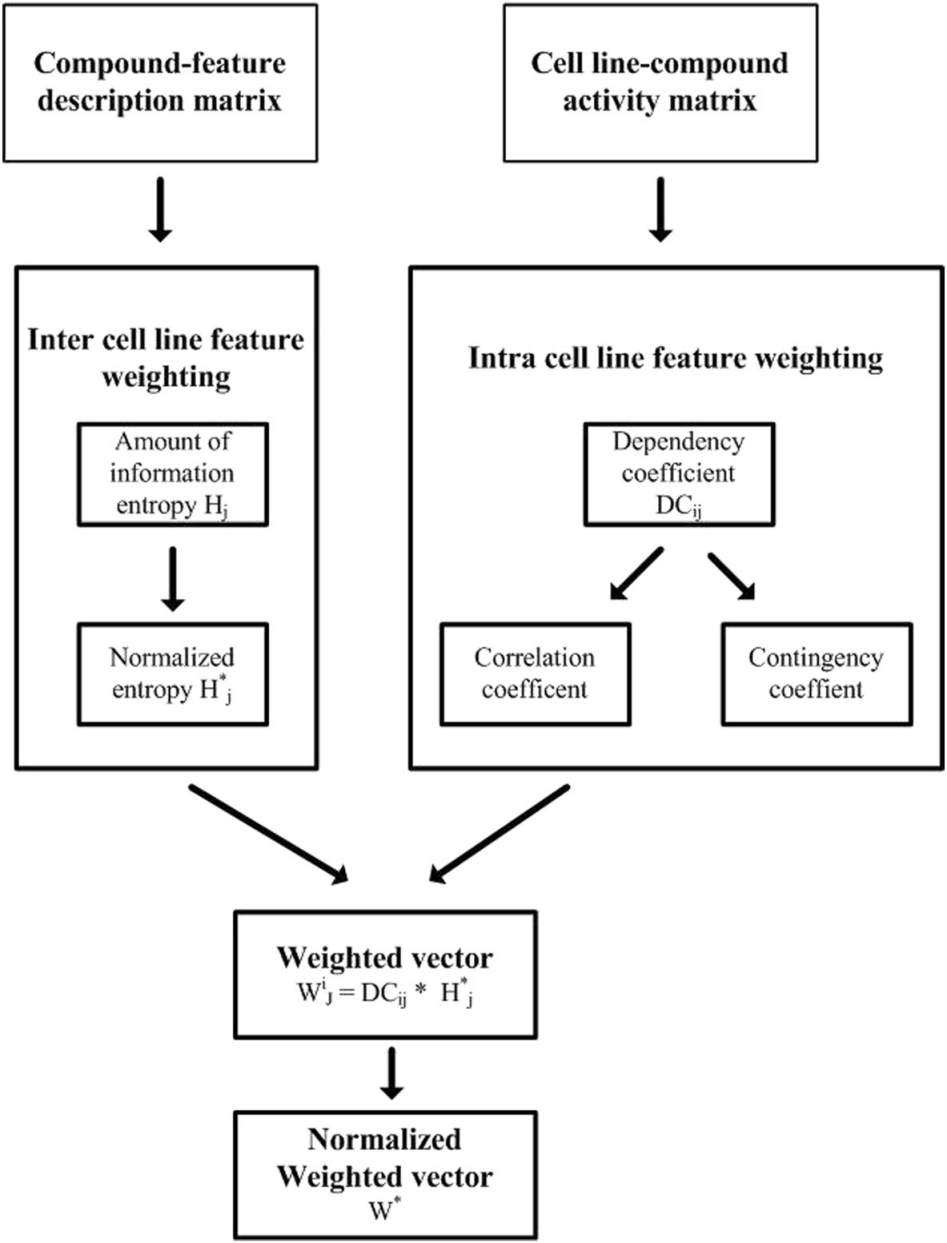
Feature weighting based on entropy and dependency measures for collaborative QSAR modeling.

A generally outline of the proposed feature selection strategy is presented in Figure 3. Detailed information can be referred to the original work [23].

## Results

We performed a comprehensive study of the collective matrix factorization based multiple cell line QSAR modeling for the inhibitors of Hedgehog Signaling Pathway as described in Section 2. In the rest of this section we present and summarize the key results from this study. The performance of our method was compared with the baseline QSAR models of liner ridge regression and SAR. Details are listed in the following.

### Performance of the collective matrix factorization based QSAR modeling

**Figure 4:**
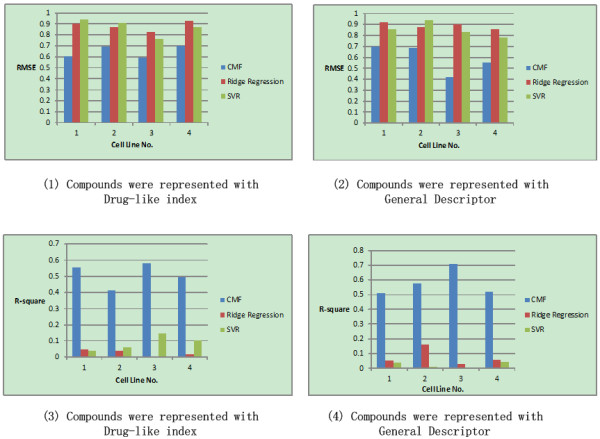
**Comparison of CMF based QSAR modeling with ridge regression and SVR on the inhibitors of Hedgehog Signaling Pathway.** The compounds are represented with Drug-like index and General Descriptor respectively. The whole data was partitioned randomly with selecting 2/3 data as training dataset and 1/3 data as testing dataset. The whole procedure was repeated 10 times and the averaged performance was calculated. Cell line No. 1–4 corresponding to BxPC-3, NCI-H446, SW1990 and NCI-H157 respectively.

**Figure 5:**
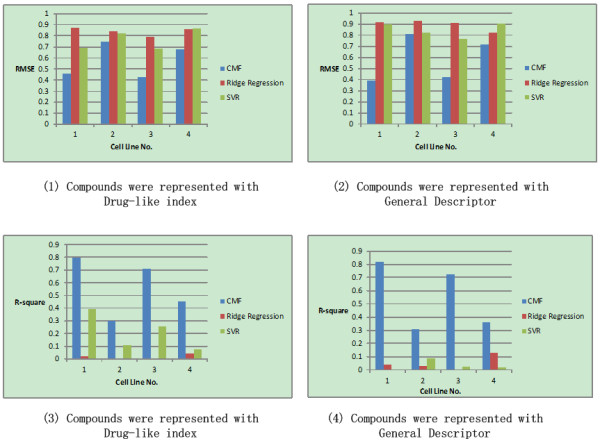
**Comparison of CMF based QSAR modeling with ridge regression and SVR on the inhibitors of Hedgehog Signaling Pathway.** The compounds are represented with Drug-like index and General Descriptor respectively. The whole data was partitioned with *Diverse subset* method. Cell line No. 1–4 corresponding to BxPC-3, NCI-H446, SW1990 and NCI-H157 respectively.

Figures 4 and 5 present average improvements achieved by the CMF based multiple cell line QSAR modeling over the baseline methods for four cell lines, with two different kinds of drug representations, i.e., general descriptor and drug-like index respectively. Figure 4 shows the performance result of the first partition strategy, where the test was carried out under certain parameter setting and with 10 times repetition by randomly selected 2/3 data as training dataset and 1/3 data as testing datasets. Figure 5 shows the second strategy, where the test was carried out under certain parameter setting with *diverse subset* to consider the data representative ability in the training and testing dataset.

From Figure 4,5, it can be seen that that the different data partition strategies actually achieve the similar performance results. For all the cell lines and all the kinds of data representation, the performance improvement of collaborative QSAR modeling was dramatic, especially for the evaluation of *R*-square. The improvement is statistically significant, with significant *p*-value measured by RMSE and *R*-square respectively. We had already noticed in our previous study [7] that under the measurement of *R*-square, the QSAR modeling results for the four cell lines with numeric compound activities were not satisfied, indicating a satisfiable QSAR modeling against single cell line individually was hard to obtain. In contrast, in our current collaborative QSAR modeling, performance against all the cell lines was improved. The significant improvement margin evaluated by *R*-square indicates that our CMF based QSAR modeling could successfully capture the correlation, rather than its absolute value of difference among the dataset as evaluated by RMSE.

**Figure 6:**
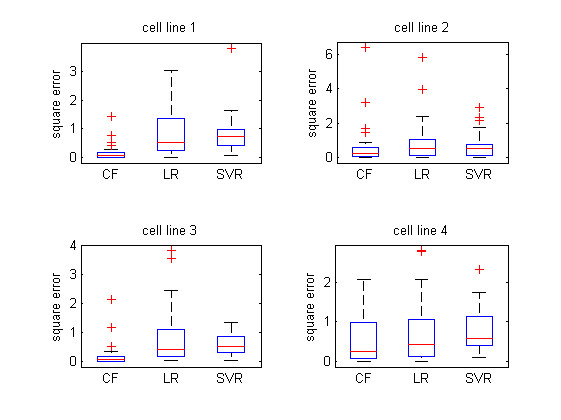
**The error distribution of different QSAR modeling visualized in boxplot.** The compound is represented in General Descriptor and the training and testing data was partitioned with *Diverse subset* method.

**Figure 7:**
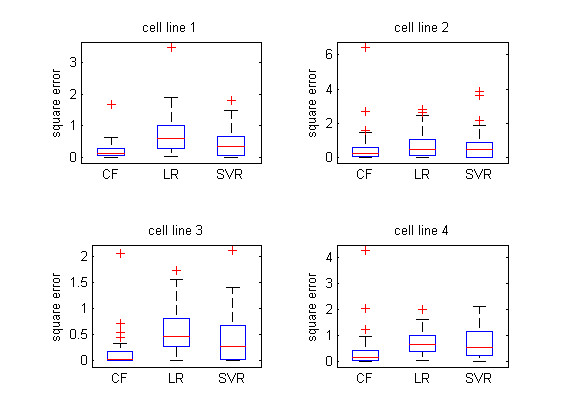
**The error distribution of different QSAR modeling visualized in boxplot.** The compound is represented in Drug-like index and the training and testing data was partitioned with *Diverse subset* method.

Besides the measurements of the average RMSE and *R*-square of different QSAR models, we also investigated their error distribution under the *diverse subset* partition strategy to give a more rigorous comparison of their performance. It can be seen from the boxplots of the error square (Figure 6–7) that for both two compound descriptions, collaborative QSAR modeling achieved the lowest error means and low variances compared to other two baselines, indicating the best prediction ability among all methods.

It should be noted that in our previous study we found that different cell lines perform differently for modeling the inhibitor affinity based on the linear regression or SVR. Particularly, only the data of NCI-H446 could produce a reasonable model by QSAR analysis, probably due to the fact that the other three cell lines may be less sensitive as HCI-H466 cells to the hedgehog signaling inhibitor [7]. Nevertheless, it can be seen from Figure 4,7 that if we combine all these data from different cell lines together under the CMF based QSAR modeling, we can greatly reduce such non-specific effects in the cell lines, and result in a reasonable QSAR modeling against all the cell lines respectively. Such improvement is attributed to the fact that the collaborative filtering based framework allows different cell line data tasks to enhance each other during the training process, which eventually makes the efficacy modeling better than those of using the datasets separately. We believe that such “collaborative” scenario for drug analysis will become more popular in the future, as more and more cell line will exist and the drug are often required to be investigated under various circumstances.

Finally, in order to evaluate whether our collaborative QSAR model is general enough for new predictions, we also checked the *domain of application* (DOA) for the model under the *diverse subset* partition strategy. The domain of application (DOA) is used to estimate the reliability in the prediction of a new compound [24] for a specific method. Those molecules fall out the domain may lead to unreliable predictions [10]. In the analysis of DOA, a value of leverage $h_{i}$ is defined in equation (11) for each chemical molecule:

(11) $$h_{i}=X_{i}^{\text{T}}{\left(X^{\text{T}}X\right)}^{-1}X_{i}$$

Where $X_{i}$ is the row-vector descriptor of the query compound,*X*is the n×k matrix containing *k*descriptor values and *n* training samples. The superscript *T* is the transpose of the matrix or vector. Generally, the warning leverage h* is fixed at 3k/n, where *n* is the number of training compounds, and *k* is the number of descriptors. When the leverage is greater than the warning leverageh*, the predicted activity is the result of substantial extrapolation of the model and, therefore, it may not be reliable and tend to be over-fitting.

**Figure 8:**
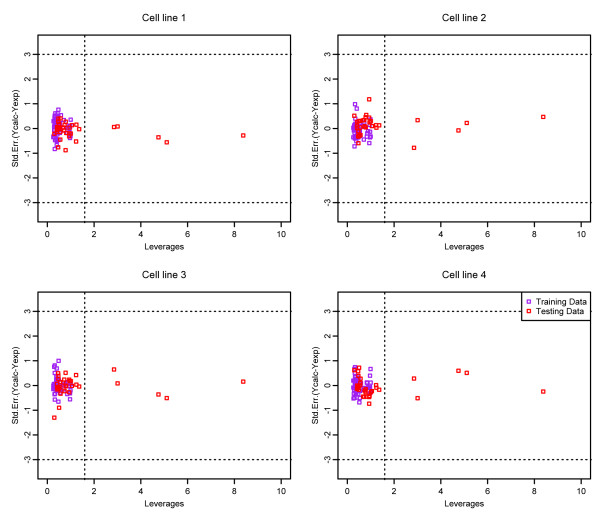
Williams plots of the collaborative QSAR model with compounds represented in general descriptor.

**Figure 9:**
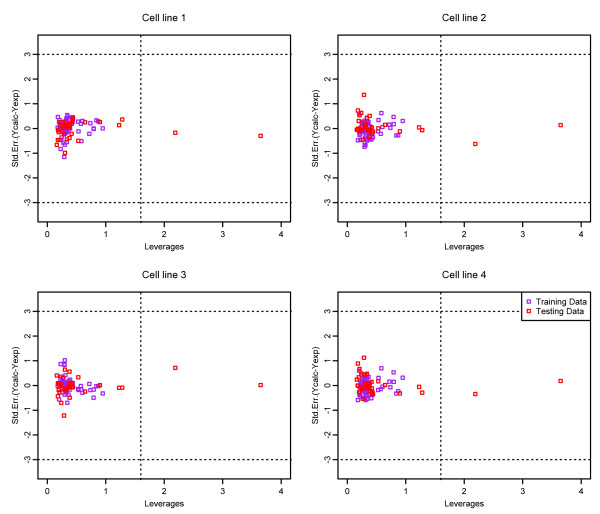
Williams plots of the collaborative QSAR model with compounds represented in drug-like index.

Based on the definition of leverage, Williams plot was used in this study to visualize the DOA of the QSAR model [10]. The Williams plot plots the standardized cross-validated residuals (RES) versus leverage values (*h*), and can be used to obtain an immediate and simple graphical detection of both the response outliers (*Y* outliers) and the structurally influential chemicals (*X* outliers) of a model. Generally, the points with their values of *Y* axis fall outside the 3*σ* line (*σ* is the standard residuals unit of the compounds) can be considered as *Y* outliers, while the points with their values of *X* axis fall outside the warning leverage h*line can be considered as *X* outliers [10]. Figures 8 and 9 represent the William plots for the four cell lines with compound representations of General Descriptor and Drug-like index respectively. It can be seen that for all four cell lines, most of the compounds fall into their corresponding application domain, which indicate that the collaborative QSAR modeling has achieved a reliable activity prediction for the compounds, and they are following a well-defined domain of applicability.

### Impact of the Regularization Parameters

**Figure 10:**
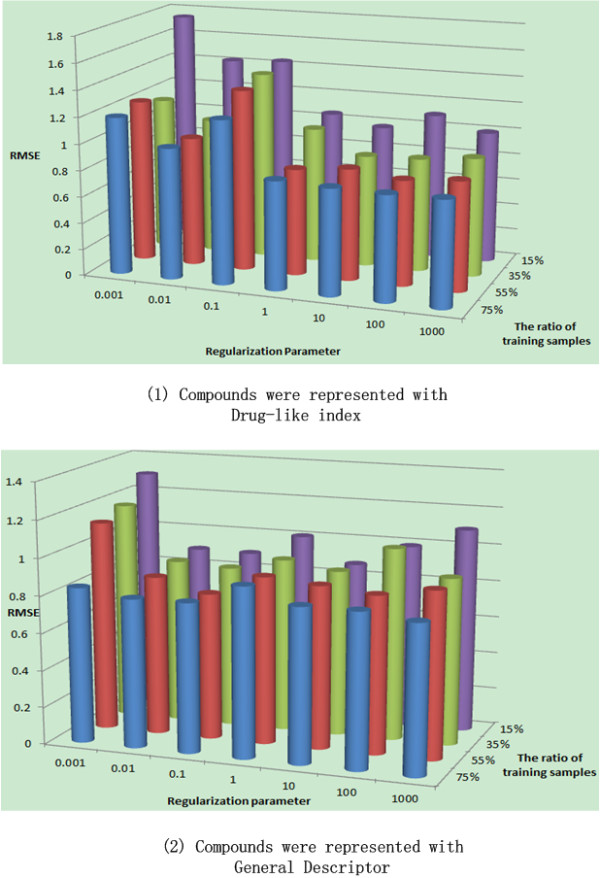
**The impact of regularization parameter and training sample ratio on the performance of collaborative QSAR modeling.** The compounds are represented with Drug-like index and General Descriptor respectively.

In this subsection, we will investigate the impact of the regularization parameters on our CMF-based QSAR modeling. We choose the values of $\lambda_{1}$ and $\lambda_{2}$ under different dimensionality of low dimensional representations and different numbers of training ratings, and plot the RMSE based on the whole four cell line data as shown in Figure 10. The tests were performed with different compound descriptions, i.e., General Descriptors and Drug-Like Index respectively. In the figure, x-axis corresponds to different value of regularization parameter (0.001, 0.01, 0.1, 1, 10, 100) while y-axis corresponds to the number of training ratios for the whole QSAR data (15 %, 35 %, 55 %, 75 %). It can be seen that (1) basically the influence of the regularization parameter is small on the performance, indicating that our proposed method is robust and insensitive to the parameters, (2) higher performance will be achieved with the larger number of training samples, which is not surprising in our study, and (3) generally the two compound description, i.e, Drug-Like index and General Descriptor, performed the same in CMF with no statistically different.

### Feature selection based on CMF for compound description among multiple cell lines

Using collaborative filtering based feature selection strategy we proposed aforementioned, we obtained the feature weighting for intra-cell line and inter-cell line for the inhibitors of Hedgehog Signaling Pathway. The former one can be used to uncover the important features in inhibitor design against a specific cell line, while the later one is used to identify common features that are important for the inhibitors against multiple cell line simultaneously. We compared the difference between these two kinds of feature weighting to provide useful clues for inhibitor modifications and improve their affinities.

In this feature selection, we used Drug-like index to represent each compound, with the total of 28 features, since it is easy to interpret biological meanings. The General descriptor feature space has been hybridized, and the original meanings of compound structure description for current features couldn’t kept. Therefore, GD will not be adopted for feature weighting here. Generally speaking, Drug-like index belongs to the category of structural descriptors. Structural descriptors can correlate with each other; some of them may be redundant. However, if they have different and significant distributions in the considered drug class, they can be used for drug-knowledge extraction and the redundant can be ignored. In our study, the descriptors maintain their identity and clearly interpretable structural significance throughout the process. A table with detailed descriptions of each drug-like index is listed in Additional file 2: Table S1.

**Figure 11:**
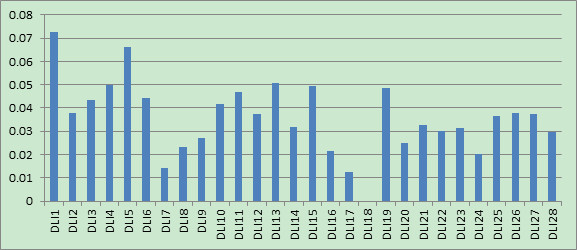
Feature weighting for inter-cell line of drug-like index.

**Figure 12:**
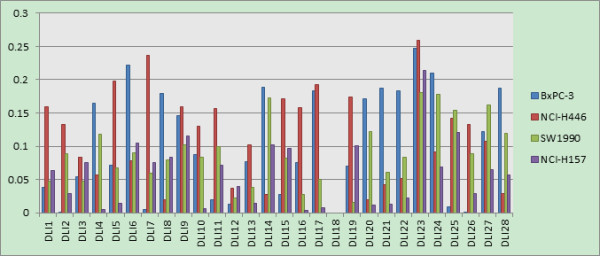
Feature weighting for intra-cell line of Drug-like index (different colors represents different cell lines).

**Figure 13:**
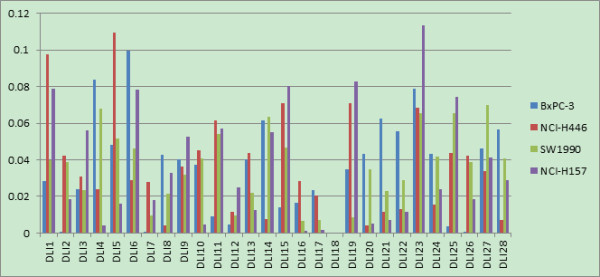
Final feature weighting with integration of inter-cell line and intra cell line information of Drug-like index (different color represents different cell lines).

The 28 feature weights for the intra-cell line, inter-cell line and the final integrated one are shown in Figures 11, 12 and 13. In all three figures, x-axis represents the Drug-like index feature ID and y-axis represents its corresponding weights. It can be seen that the final integrated feature weighting is different from that of intra-cell line. Moreover, the inter-cell line feature weighting can be viewed as an efficient way to identify the potential features important for multi-target inhibitors of Hedgehog Signaling Pathway. We provide our insights about inhibitors design based on these figures:

1) As shown in Figure 11, the features of ‘# of non-H’ (DLI1), ‘# of non-H polar bonds’ (DLI5) and ‘# of 2-degree cyclic atoms’ (DLI13) were ranked top 3. These findings indicate that the volume of the molecular, the polar of the molecular and the cyclic degree of the molecular are the most important features for the design of multi-target inhibitors of Hedgehog Signaling Pathway. Our findings are actually consistent with the empirical rules for lead compound optimization, which use these three elements to determine their activities.

2) We can see from Figure 11 that the feature ‘# of cap fragments’ (DLI23) was also important when the multi-cell line inhibitors were designed. This is consistent with the empirical rule, which changes the substituent group (functional group) in order to improve the inhibitor activity. However, compared with Figure 12, it can be seen that the importance of this feature for multi-cell line inhibitor design is not as much significant as that for individual cell lines. This could be explained that, although this feature is important for individual cell line inhibitor, their activity improvement directions may be inconsistent, thus reducing its importance when multi-cell lines are confronted.

3) All three figures (Figure 11, 12, 13) have shown that the weight for the feature of '# of 3-level bonding patterns' (DLI18) is 0. This is probably due to the following two reasons:a) All compound samples in our study are lack of this feature, and b) this feature is not considered in most of the in-silico compound optimizations.

## Discussion

### Comparison of CMF based QSAR modeling with other collaborative QSAR modeling

Although the CMF based QSAR modeling was investigated in our study, we do realize the existence of other QSAR modeling with integrated information, and we call such models as the “collaborative” QSAR modeling, like the neural network based [15][25-27] and multi-task learning based [9][10] models, as well as the proteochemometrics modeling (PCM) [28][29]. In order to further uncover the characteristics of such collaborative QSAR modeling, we discuss our CMF based method with the aforementioned methods on our multiple cell line QSAR modeling for the inhibitors of Hedgehog Signaling Pathway.

### Neural network based collaborative QSAR modeling

As we mentioned above, Erhan etc. proposed a neural network based collaborative QSAR modeling for drug discovery [15]. This is one of the first attempts to construct an efficient procedure for integrating multiple drug target information at a time by extending standard multi-layer neural networks. Basically, neural networks provided an ideal test bed for implementing collaborative QSAR modeling: the simplest of such form was to create a shared hidden layer that is trained in parallel for all the learning tasks. In this case, the training procedure would be done on all the tasks (in our study it will be all the cell line QSAR data) in parallel. Because the structure of the network includes a shared layer (weight matrix), it is possible for so-called “shared internal representations” to develop and to be learned.

Specifically, in our multiple cell line QSAR modeling for the inhibitors of Hedgehog Signaling Pathway, we used a 10-cross fold validation schema to test our data from 4 cell lines in this neural network model. The weights from input layer to hidden layer as well as from hidden layer to output layer for the network will be learned through the back propagation (BP) algorithm. Our in-house test indicated that the neural network based collaborative QSAR modeling was comparable to CMF based QSAR modeling, with no surprisingly better than the single QSAR modeling (Results are not shown here).

### Multi-task learning based collaborative QSAR modeling

Neural network can be viewed as a specific form of multi-task learning. Multi-task learning has been developed for those situations where multiple related learning tasks are to be accomplished together. When explicit or hidden interrelationship among the tasks can be exploited [9][10], multi-task learning is more effective than learning each task independently. The intuition underlying the framework is that the multiple related tasks can benefit each other by sharing the data and features across the tasks, and thus boosting the learning performance of each single task [30]. It also provides an efficient mechanism for cross-task feature selection, thus uncovering the common dominate features for all the tasks simultaneously. Our group has successfully applied multi-task learning in QSAR modeling with specific study of HIV and HCV inhibitors [9][10]. Basically, assume that the datasets contain *N* tuples, $z_{i}=({\text{x}}_{i},y_{i},k_{i})$ for i = {1…*N*}, where ${\text{x}}_{i}\in {\text{R}}^{d}$ is the drug descriptor, and $k_{i}\in${1…*M*} is the indicator corresponding to the example ${(\text{x}}_{i},y_{i})$. The *M* tasks correspond to *M* different cell lines or drug targets. A critical issue in this collaborative QSAR modeling is to learn a set of sparse functions across these tasks for drug activity regression. This is commonly achieved by learning *M* linear regressions of the form $w_{k}^{T}x$, with the following square loss function is adopted (other loss function can also be applied):

(12) $$l_{s}(z,W)={\left(y-{\text{w}}_{k}^{T}\cdot \text{x}\right)}^{2}$$

wherez=(x,y,k), *W*$=[{\text{w}}_{1},{\text{w}}_{2},\ldots ,{\text{w}}_{M}]\in {\text{R}}^{d\times M}$ and $W^{j}$ be the *j*th row of *W*.

In the multi-task learning framework, *W* can be optimized and calculated by enforcing the joint sparsity across different tasks with adding the different norm of the matrix *W* to the square loss function, which leads to only a few non-zero rows of *W*.

The relationship between collaborative filtering and multi-task learning has been discussed in previous studies [30]. The multi-task learning model is closed related to the multiple response regression models [31]. Multiple response regression is the task of estimating several response variables using a common set of input variables. In general, both multi-task learning and multiple response regression can be used to find the correlation between different tasks, and thus improve the single task learning. Such an approach have many potential applications in various areas, interested readers may be referred to the paper [31]. It should be noted that in the multi-task learning framework, the samples for different tasks should not be identical. In general, the less overlap of the samples containing across different tasks, the more prediction ability of each task. This idea is related to another interesting algorithm, transfer learning [32], whereas multi-task learning can be categorized into this area and the information between different tasks is expected to “transfer” from each other to boost the performance of individual task.

For the particular data in our multiple cell line QSAR modeling for the inhibitors of Hedgehog Signaling Pathway, it can be seen that the drug samples for all the cell line are totally identical, thus it is unnecessary to use multi-task learning in the collaborative QSAR here. Nevertheless, if non-identical samples for multiple cell line exist, multi-task learning will be a good choice for collaborative QSAR modeling with integrating of different data sources.

### Proteochemometric Modeling

**Figure 14:**
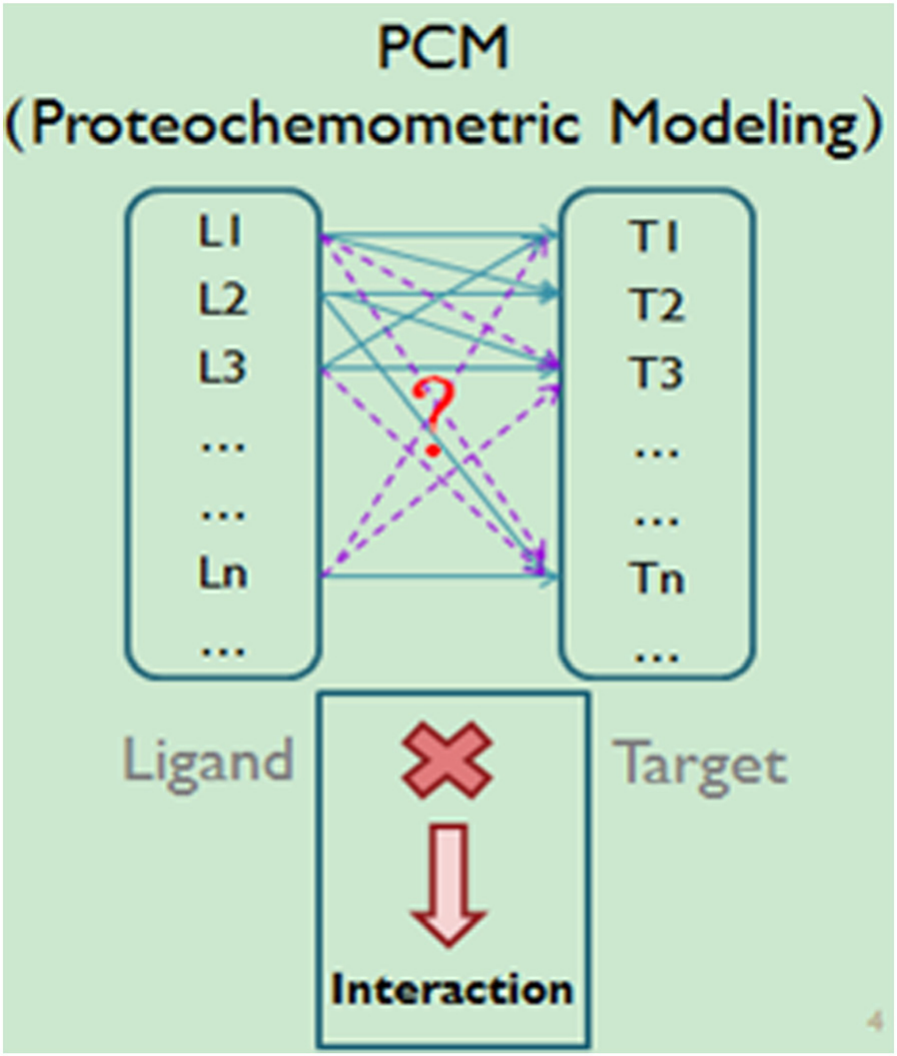
Illustration of proteochemometric modeling.

Proteochemometric modeling (PCM) is presented based on the similarity of a group of ligands and a group of targets, to the extent that PCM models the so-called ligand-target interaction space [28][29]. Like QSAR modeling, the PCM model is constructed based on chemical descriptors that describe the compound data set and it introduces an additional term, a descriptor of the protein - target interaction (Figure 14). Therefore, a PCM model is constructed on both ligand and target similarity, and it can be regarded as an extension of conventional QSAR modeling, which models the relationship between multiple compounds and targets simultaneously. PCM is intrinsically the most similar to our collaborative filtering based QSAR modeling among all others. PCM explicitly requires the target information as well as the protein-target interaction descriptions. Whereas in our collaborative filtering based QSAR modeling, these two kinds of information are implicitly embedded in one computational schema. From this point of view, our model is more flexible and extendable. Since in our multiple cell line QSAR modeling for the inhibitors of Hedgehog Signaling Pathway, there is no explicit target information available, we cannot use PCM for the QSAR modeling. Large-scale ligand-target relationship study and comparison between collaborative filtering based methods and PCM still remain to be an interesting and useful topic for the future study.

## Conclusions

In this study, an efficient collaborative QSAR model for inhibitors of Hedgehog Signal Pathway from multiple cell lines was proposed. The model is derived from the area of information retrieval in social network, i.e. collaborative filtering, and its performance is well demonstrated and explained in our study. By applying this elegant computational model, we successfully addressed two issues remained in our previous study, i.e., (1) The information among multiple cell lines can be integrated to boost the QSAR results, rather than single cell line QSAR modeling. Our extensive experiments indicated that the performance is remarkable compared to other single cell line QSAR methods. (2) A novel feature selection strategy under such collaborative environment was proposed, which can be used to derive the commonly important features related to the entire given cell lines, while meantime presenting their specific contributions to a specific cell-line. Based on the results of feature selection, we presented several ways of chemical modifications which will likely improve the compound affinity towards multiple targets in the Hedgehog Signal Pathway simultaneously. In summary, our study provides useful clues for multiple cell line/targets QSAR modeling when the cell line or target information among a related pathway exist. The proposed collaborative model with the feature selection strategy here is efficient, robust, flexible, and can be easily extended to model large-scale multiple cell line/QSAR data.

## Competing interests

The authors declare that they have no competing interests.

## Authors' contributions

QL and JG carried out the designing of the whole computational algorithm and drafted the manuscript. JG and DC were responsible for the algorithm implementation. DC, VZ and RZ were responsible for the algorithm design. QL conceived the study and participated in the design and coordination of the analyses. All authors read and approved the final manuscript.

## Supplementary Material

Additional file 1 The data and scripts for collaborative QSAR modeling. Click here for file

Additional file 2 **Table S1.** Detailed explanations of each Drug-like index. Click here for file
