# Supplementary material for: Integrated QSAR study for inhibitors of hedgehog signal pathway against multiple cell lines:a collaborative filtering method
Source: BMC Bioinformatics. 2012 Jul 31;13:186. doi: 10.1186/1471-2105-13-186 (PMC3522553; doi:10.1186/1471-2105-13-186)
Supplement: Additional file 2 — Table S1. Detailed explanations of each Drug-like index. [file 1471-2105-13-186-S2.docx]

Table S1: Detailed explanations of each Drug-like index

| Drug-like index ID | Descriptions |
| --- | --- |
| DLI(01) | '# of non-H' |
| DLI(02) | 'total SSSR size' |
| DLI(03) | 'degree of cyclization' |
| DLI(04) | '# of rotatable bonds' |
| DLI(05) | '# of non-H polar bonds' |
| DLI(06) | '# of carbons in cap fragments' |
| DLI(07) | '# of N with # of H > 0' |
| DLI(08) | '# of hydroxyl groups' |
| DLI(09) | '# of H-bond donors' |
| DLI(10) | '# of H-bond acceptors' |
| DLI(11) | '# of N and O atoms' |
| DLI(12) | '# of 2-degree acyclic atoms' |
| DLI(13) | '# of 2-degree cyclic atoms' |
| DLI(14) | '#of 3-degree acyclic atoms' |
| DLI(15) | '# of 3-degree cyclic atoms' |
| DLI(16) | '# of 1-level bonding patterns' |
| DLI(17) | '# of 2-level bonding patterns' |
| DLI(18) | '# of 3-level bonding patterns' |
| DLI(19) | '# of fragments' |
| DLI(20) | '# of aromatic systems' |
| DLI(21) | '# of cyclic fragments' |
| DLI(22) | '# of linkers' |
| DLI(23) | '# of cap fragments' |
| DLI(24) | 'maximum SSSR size' |
| DLI(25) | 'maximum cap fragment size' |
| DLI(26) | 'total number of 3-8 membered rings' |
| DLI(27) | 'total number of 3to8 saturated rings' |
| DLI(28) | 'total number of 3to8 unsaturated rings' |
